# Fabrication and Electrical Characterization of Dot Capacitors for Cold Field Emission Applications

**DOI:** 10.1021/acsomega.4c10081

**Published:** 2025-03-14

**Authors:** Ammar Alsoud, Adel A. Shaheen, Alexandr Knápek, Saleh R. Al-Bashaish, M D (Assa’d) Jaber Ahmad, Marwan S. Mousa, Dinara Sobola

**Affiliations:** 1 Central European Institute of Technology, Brno University of Technology, Purkynova 656/123, Brno 61200, Czech Republic; 2 Department of Physics, Faculty of Electrical Engineering and Communication, Brno University of Technology, Technická 2848/8, Brno 61600, Czech Republic; 3 Department of Physics, Faculty of Science, The Hashemite University, P.O. Box 330127, Zarqa 13133, Jordan; 4 Institute of Scientific Instruments of the Czech Academy of Sciences, Královopolská 147, Brno 61200, Czech Republic; 5 Department of Microelectronics,Central European Institute of Technology Brno University of Technology, Technicka 3058/10, Brno 61600, Czech Republic; 6 Department of Allied sciences, Faculty of Arts and Sciences, Al-Ahliyya Amman University, Amman 19328, Jordan; 7 Department of Basic Medical Sciences, Faculty of Medicine, Aqaba Medical Sciences University, Aqaba 77110, Jordan; 8 Department of Renewable Energy Engineering, Jadara University, Irbid 21110, Jordan; 9 Institute of Physics of Materials, Czech Academy of Sciences, Žižkova 22, Brno 61662, Czech Republic

## Abstract

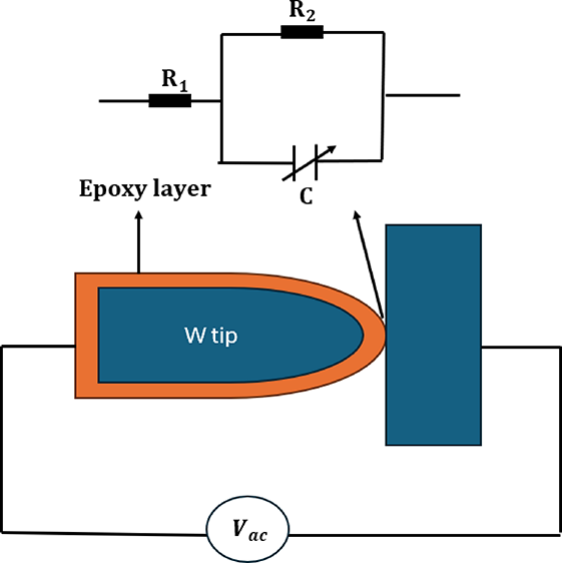

The aim of this work was to study the dielectric properties of dot capacitors composed of a microtip coated with a thin layer of epoxy resin bonded to a steel plate. Two microtips with radii ranging from 3 to 5 μm were fabricated via electrochemical etching and coated with an epoxy layer 27–35 μm in thickness. The microtips were characterized by scanning electron microscopy-energy dispersive X-ray spectroscopy (SEM-EDS). This study showed that composite cold-field emission emitters behave as dot capacitors. The real and imaginary parts of the impedance and permittivity, along with the direct and alternating conductivities, activation energies, and hopping energies, were examined. These evaluations were conducted at temperatures of 30, 45, 60, 75, and 90 °C, with a frequency range of 1 to 10^6^ Hz using impedance spectroscopy. The results indicated that both the impedance and electrical permittivity decreased slightly with increasing temperature, whereas the AC conductivity was independent of temperature. Additionally, a decrease in the activation and jump energies was observed as the thickness of the epoxy layer increased. The low values of the activation and hopping energies facilitated electron transport through the epoxy layer. The modified hopping model also provides an explanation for the conduction mechanism through the epoxy layer. The Nyquist plot shows that the capacitance decreased with increasing temperature. A slight increase in relaxation time was also observed, indicating the onset of conductive pathway formation. These findings contribute to a better understanding of the capacitance of the composite emitters and the formation of conductive pathways.

## 1 Introduction

A composite emitter is a sharp metal tip coated with a thin layer of insulating film or oxide.^1,2^ These emitters are widely used for cold field emission applications under high vacuum conditions with the goal of achieving a stable high emission current when several hundred volts are applied. The coating layer plays a crucial role in protecting the sharp tip from ion bombardment and focusing the emitted electrons onto a single bright spot.^2,3^ Composite emitters are commonly employed in various electron microscope applications such as electron microscopes,^3^ semiconductor inspection tools,^4^ and transistors.^5^

Electron emission has been extensively studied, both theoretically and experimentally. The mechanism of electron emission from sharp tips has been thoroughly investigated in the literature, primarily using the Fowler-Nordheim and Murphy-Goode equations as well as their optimizations, which aim to explore the relationship between the emission current and applied voltage.^6−10^ However, in most cases, the composite emitters did not undergo orthodoxy testing.^11−14^ An orthodoxy test can help confirm whether electron emission data are consistent with classical or ‘orthodox’ field emission theory, which is based on Fowler-Nordheim (FN) and Murphy-Goode (MG) theory. These tests assessed whether the field emission followed the expected trends, such as the linear relationship in the FN and MG plots.^15^

By contrast, Zhou et al.^16^ developed a quantitative analytical solution for field emission from dielectrically coated cathode surfaces by solving the one-dimensional (1D) Schrödinger equation, accounting for the double potential barrier introduced by the coating layer. Despite these insights, this model faces practical challenges because of the difficulty in accurately calculating the dielectric constant of composite emitters.

Several experimental hypotheses have been proposed to explain the mechanism of electron emission through dielectric layers. Latham and Mousa^1^ introduced a model titled ″Hot Electron Emission from Composite Metal–Insulator Microcathodes.″ Additionally, Knapek et al. proposed the ″Volcanic Eruption″ model. Knapek et al.^17^ examined the effects of noise on electron emitters by focusing on cold-field emitters comprising a sharp metal tip coated with a thin epoxy layer. Ji et al.^18^ proposed a self-compensating electron emission mechanism for bulk graphene to explain its enhanced field emission performance. The most recent hypothesis is that the trap-rich capacitor model proposed by Alsoud et al..^19^

Despite these explanations, research on the changes in the electrical properties of the dielectric layer in composite emitters has been limited because of several challenges: the nanoscale size of the emitters, thinness of the coating layer (also at the nanoscale), and absence of a suitable sample holder, as composite emitters are highly sensitive and prone to significant measurement errors. To address these issues, we developed a custom sample holder to accommodate the required sample size. In addition, large-radius emitters (Mirco Scale) were employed to enhance the accuracy of the results. In this work, the term ″Dot capacitor″ (DCa) refers to a sharp metal tip coated with a thin dielectric layer, mounted on a steel plate.

To better understand the mechanisms of conduction, several studies have analyzed the solid-state interaction at high temperatures in depth. Halder et al.^20^ studied the dielectric properties of *MoO*_3_ doped *V*_2_*O*_3_ – *ZnO* – *CuO* glass nanocomposites. The incorporation of *V*^2+^ and *Mo*^6+^ions was employed to enhance electrical conductivity. The observed conductivity was analyzed using a correlated barrier-hopping model. Poddar et al.^21^ investigated structure-dependent dielectric properties of AgI-doped glass systems. They examined various aspects of the conduction process by analyzing the transport of charge carriers in glass conductors across different temperature ranges and frequency windows. This study also investigated the conduction mechanisms and explored the short- and long-range ordering of charge carrier relaxation to elucidate the relationship between the microstructure and conduction processes in AgI-doped glass systems. In a separate study, Assayed et al.^22^ investigated the electrical characteristics and dielectric behavior of cadmium phosphate glasses doped with NaCl. The researchers provided comprehensive insights into the changes in the conductivity of the material with respect to the frequency of the applied electric field and the changes in temperature and anesthetic concentration. The conduction mechanism, involving both ionic and electronic pathways, was elucidated. In addition, several studies have characterized the conduction mechanism in nanocomposite systems.^23−28^

This study is the first to investigate the electrical properties of epoxy layers in composite emitters using the point capacitor concept. The objective of this study is to characterize the capacitive behavior of composite emitters and elucidate the conduction mechanisms within the epoxy layer. This was achieved by analyzing the electrical properties using impedance spectroscopy over a temperature range of 30 to 90 °C to focus on the thermal activation of charge carriers. The measurements were conducted across a frequency range of 1 −10^6^ Hz, which is particularly relevant for transistor applications. The study further revealed that nonuniform coating thicknesses on the emitter surface could induce deformations in the epoxy chains at the tungsten interface. This leads to the formation of polarons and traps, which contribute to the variations in the maximum barrier height. These variations are hypothesized to significantly influence the conduction process. The results indicate that conductivity is largely temperature-independent, whereas polaron formation notably affects electron storage. A modified correlated barrier hopping (CBH) model was identified as the most appropriate theoretical framework for describing the conduction process. Additionally, the relaxation times were observed to decrease with increasing temperature, suggesting accelerated charge carrier mobility, which enhances the understanding of the mechanisms of electron emission and the formation of conductive pathways in such systems, leading to further optimization in industrial and technological applications.

## 2 Methodology

### 2.1 Sample Preparation

**Figure 1:**
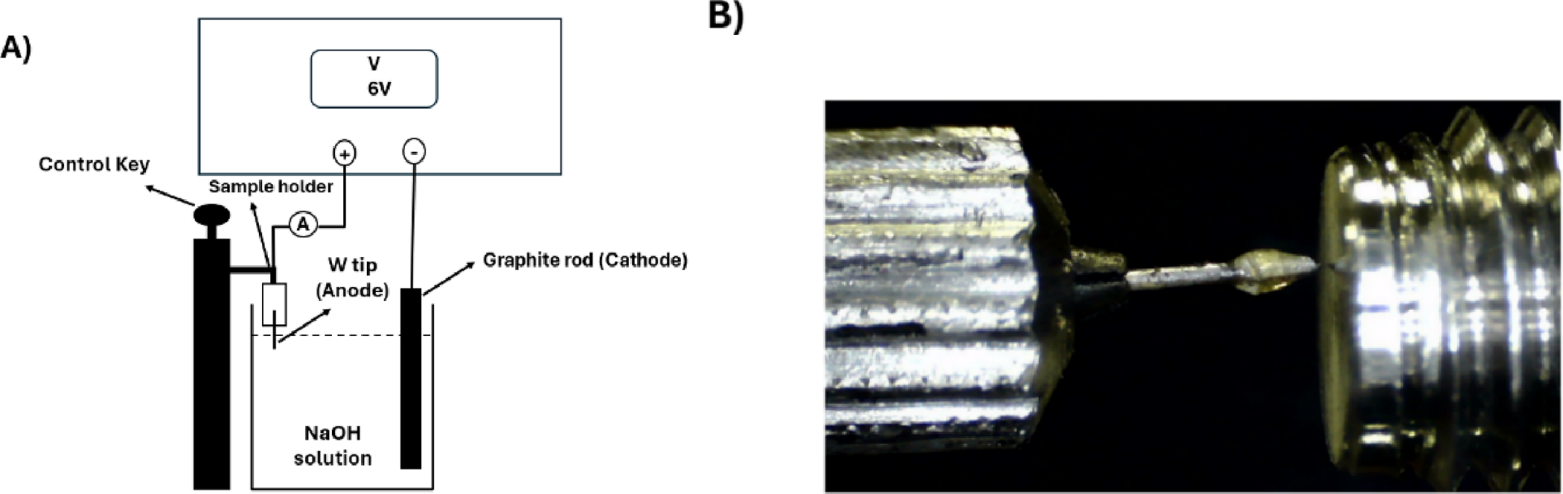
(A) Schematic diagram of electrochemical etching setup. (B) Dot capacitor.

The tungsten (W) tip was fabricated using electrolytic etching. A tungsten wire, 0.35 mm diameter and 1 cm length, was immersed in an electrolyte solution composed of 250 mL water and 25 g NaOH. A tungsten wire was used as the anode and a graphite rod was used as the cathode. Both electrodes were submerged in a NaOH solution and connected to a DC power supply set at 6 V. The etching process lasted for approximately 10 min. When around 0.4 cm of the tungsten wire was immersed in the NaOH solution, the initial etch current was approximately 30 μA, gradually decreasing as the wire thinned over time, continuing until the lower portion of the wire dropped off. Figure 1a shows a schematic of the electrochemical etching setup. As the bottom section decreased, the resistance of the etching circuit increased sharply, prompting rapid shutdown of the DC voltage source. The timing of this shutdown significantly affected the sharpness of the tip. After etching, the tungsten wire was carefully removed from the solution and immediately cleaned by briefly dipping it in alcohol and distilled water. Subsequently, the tip was further cleaned by immersion in Hydrofluoric acid (HF) for 10 min. This process produced tungsten tips with radii in the range 3–5 μm.

Epoxylite E478 (E-478) sourced from Elantas (Wessel, Germany) was used for coating. The microtip was mounted on a sample holder that allowed vertical movement, enabling it to be lowered into an epoxy resin beaker. Maintaining the tip perpendicular to the surface of the epoxy resin ensured uniform coating on the tip apex. The tip was slowly immersed in the resin 10 times to minimize defects, such as air bubbles in the coating layer.

To stabilize the coating on the tip, it was cured in two stages. First, the coated tip was heated for 30 min at 80 °C for solvent evaporation, followed by an additional 30 min at 180 °C to complete the curing process. Once sample preparation was complete, a spot capacitor was formed by placing the composite tip in close proximity to a steel plate. The contact between the composite tip and plate was closely monitored using a mini microscope to ensure proper contact. Figure 1b illustrates the setup of the dot capacitor.

### 2.2 Impedance Spectroscopy

In this study, impedance spectroscopy was used to measure the impedances of the samples. The system comprised a computer-controlled Impedance Gain/Phase Analyzer (frequency response analyzer, FRA), Impedance Interface, sample holder, and a temperature-controlled oven. The computer controlled the FRA and Impedance Interface to facilitate the impedance measurements of the test sample. A capacitor was fabricated by placing a thoroughly cleaned and polished sample between two parallel Cu electrodes. The capacitor was then placed in a temperature-regulated oven and connected to a Solartron-1260 Impedance/Gain Phase Analyzer equipped with a dielectric interface (model 129, USA). AC impedance measurements were performed at specific temperatures within a frequency range of 1–10^6^ Hz and a temperature range of 30 to 90 °C. Z-View software was employed to fit the resulting impedance plots, enabling the derivation of permittivity and conductivity parameters, as well as Nyquist plots.

## 3 Characterization

### 3.1 SEM-EDS

**Figure 2:**
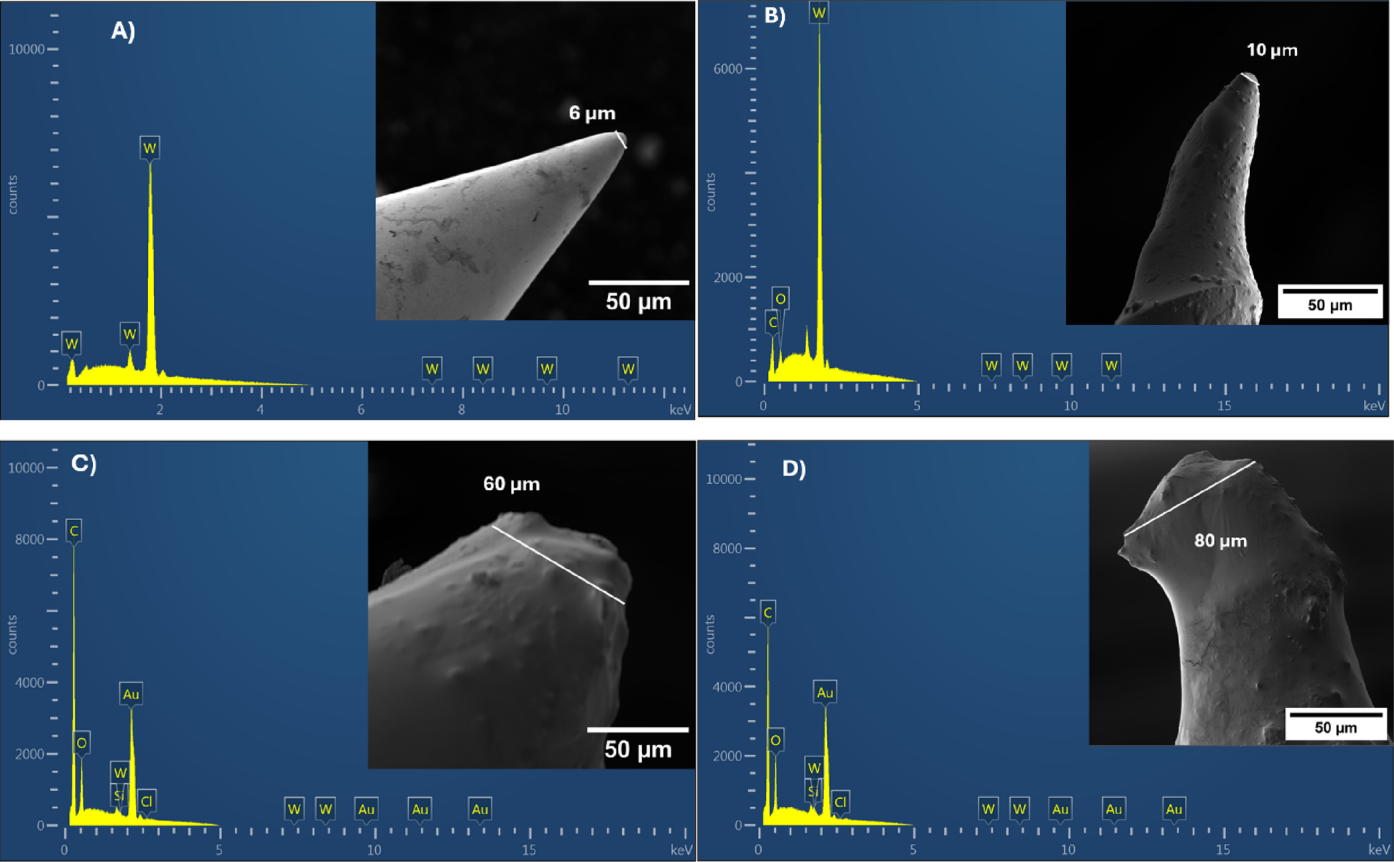
SEM micrograph and EDS spectra of (a) uncoated W1 tip, (b) uncoated W2 tip, (c) W composite tip 1, and (d) W composite tip 2.

In this study, the compositions of the W1 and W2 tips were analyzed before and after coating using scanning electron microscopy with energy-dispersive X-ray spectroscopy (SEM-EDS) (MIRA-TESCAN, Czech Republic). This analysis aimed to determine the radius of the W tips and their purity following the cleaning process. Additionally, the thickness of the epoxy coating layer and its components are illustrated in Figures 2a, 2b, 2c, and 2d, which display the SEM micrographs and EDS spectra of all samples before and after coating. The measurements were performed at the apex of the tungsten tip. Figures 2a and 2b show the radii of tips W1 and W2, with tip W1 measuring 3 μm and tip W2 measuring 5 μm. The EDS spectrum for tip W1 indicated high purity as it was free from impurities and displayed peaks corresponding to W. In contrast, carbon and oxygen peaks were detected in the EDS spectrum of tip W2, which were attributed to the components of the carbon tape used to stabilize the samples. Figures 2c and 2d depict the radii of composite tips W1 and W2, which were approximately 30 and 40 μm, respectively. The thickness of the epoxy layer at the apex of each tip was calculated by subtracting the radius of the composite tip from that of the uncoated emitter: Consequently, the thickness of the epoxy layer for the composite tip W1 was 27 μm, whereas that for the composite tip W2 was 35 μm. The EDS results indicate that the components of epoxy included carbon, oxygen, and chlorine. Gold was utilized as a coating material for the SEM-EDS measurements, and its components have been examined in detail in several previous studies.^29−32^

## Results and Discussion

### 4.1 Real and Imaginary Parts of Impedance

The complex impedance can be expressed in terms of its real (*Z*^′^) and imaginary (*Z*^″^) components, as follows:^33,34^
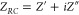
1Where , both of *Z*^′^ and *Z*^″^ can be written as
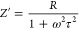
2
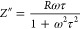
3where ω is the angular frequency, R is the resistance, and τ is the relaxation time, which is the duration required for the conduction process. Polarization refers to the time required for the system to return to equilibrium after the poles have been polarized. For the relaxation time associated with the entire conduction process, τ can be calculated from the relaxation peak at a higher frequency (*f*_*max*_) in *Z*^″^ vs *f* using the following formula:^33,34^
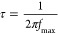
4

**Figure 3:**
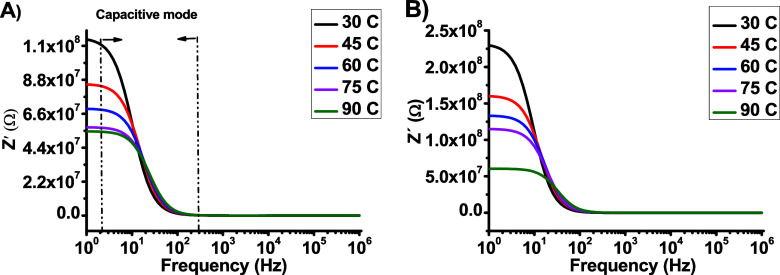
Frequency dependence of *Z*′ for (a) DCa1 and (b) DCa2.

**Figure 4:**
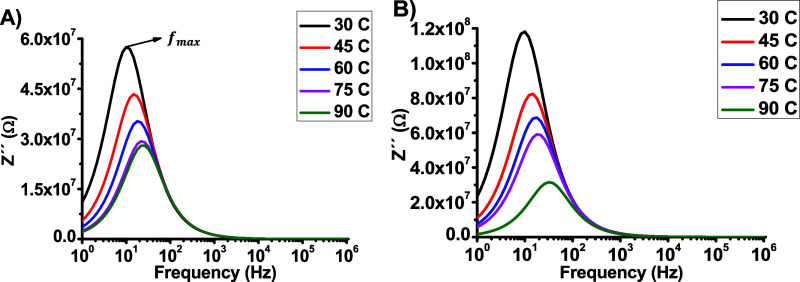
Frequency dependence of *Z*″ for (a) DCa1 and (b) DCa2.

Figures 3a and 3b show the frequency dependence of *Z*^′^ for DCa1 and DCa2, respectively, while Figures 4a and 4b show the frequency dependence of *Z*^″^ for both samples. By comparing Figures 3a and 3b, it is evident that the impedance of sample DCa2 is higher than that of sample DCa1, which can be attributed to the increased thickness of the coating layer. Additionally, it can be observed that as the temperature increased, the values of *Z*^′^ decreased, reflecting the semiconducting behavior of the epoxy.

The frequency dependence of *Z*^′^ can be divided into three distinct regions. In the first region (1–10 Hz), the values of *Z*^′^ remained frequency-independent, indicating the resistive behavior of the samples. This behavior is due to the presence of a displacement current created by localized charges bound within the material. These charges are associated with different polarization mechanisms, including electronic, ionic, directional, and space-charge polarization.^22^ As the temperature increased, this region expanded, indicating the release of additional polarization and an increase in the displacement current. At the same time, many vacancies and traps within the material capture electrons originating from the Fermi level of tungsten, leading to this region being referred to as the ‘charge region’.

In the second region (10^1^- 10^2^ Hz), the values of *Z*^′^decreased rapidly with increasing frequency, indicating the capacitive nature of the samples in this frequency range.^35^ This region is important in this study, as it is where conductive channels are formed, and the conduction current, which is the current caused by the movement of moving charges within the material and is mainly responsible for dielectric losses. However, as the temperature rises, the capacitive nature decreases owing to thermal activation, promoting the transfer of electrons. Toward the end of this phase, a switch-on phenomenon, which is well-documented effect in field emission is expected to occur. At this stage, when a specific voltage is applied, the electrons traverse the conductive paths, resulting in the emission of an electric current exceeding 1 μA.^12^ Finally, in the third region (frequencies greater than 10^3^ Hz), the values of *Z*^′^ converge regardless of the temperature, which is attributed to the diminishing effect of the space charge polarization at high frequencies.^36^ In addition, increasing the temperature of the sample led to a decrease in the *Z*^′^ values, which can be attributed to the easy transport of free charges in the epoxy layer.

According to Koop’s theory, charge carriers are localized at the grain boundaries at high frequencies. This suggests that electrons accumulate at the apex of the tip, enhancing their emission and concentrating them at a single point. This phenomenon is particularly significant for applications in devices such as transistors and atomic force microscopes (AFM), where precise electron localization is critical.^37−40^

Figures 4a and 4b show that the values of *Z*^″^decrease with increasing temperature, indicating a reduction in the bulk resistance of the material. This behavior can be attributed to the increase in the number of mobile charge carriers. Additionally, it can be observed that the relaxation peak decreases in amplitude and shifts to higher frequencies as the temperature rises. This shift characterizes both the type and strength of the thermal relaxation process, which may be due to the presence of electrons and/or immobile species at lower temperatures, and defects or vacancies at higher temperatures.^22,35,41^

**Figure 5:**
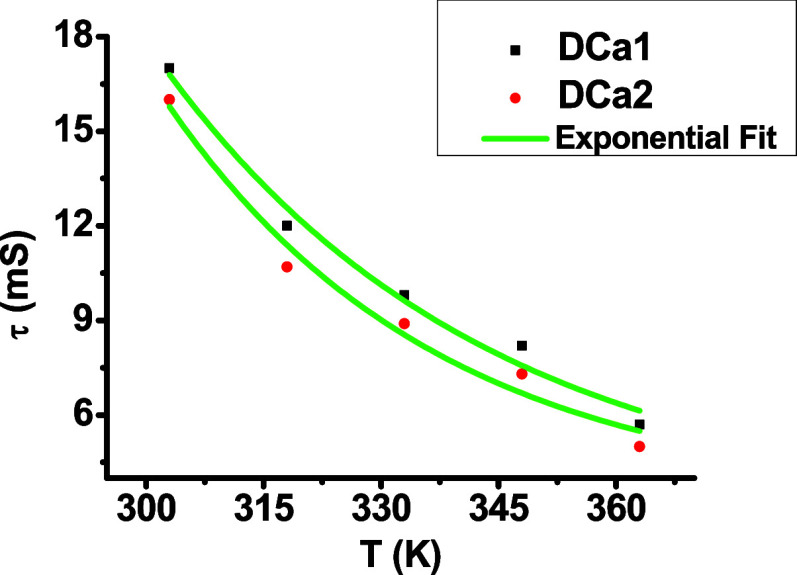
Variation of relaxation time with temperature for a dot capacitors.

As shown in Figures 4a and 4b, it is observed that the amplitude of the curves decreases with increasing temperature and the *f*_*max*_ is shifted toward higher frequencies, indicating that the relaxation time decreases with increasing temperature, indicating the thermal activation process. This indicates the presence of electrical processes in the material as the relaxation times increase. The frequency region lower than *f*_*max*_, defines the range where charge carriers are mobile over long distances, whereas for frequencies higher than *f*_*max*_, the charge carriers appear to be confined to potential wells, limiting their mobility to short distances.^42^Figure 5 illustrates the relationship between the relaxation time and temperature for the dot capacitors, where the relaxation times fit the Arrhenius equation well.^29,43^

### 4.2 Real and Imaginary Parts of the Permittivity

The complex permittivity ε̂ (ω) written in formal into real ε^′^(ω) and imaginary ε^″^(ω) components^44^:
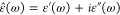
5

**Figure 6:**
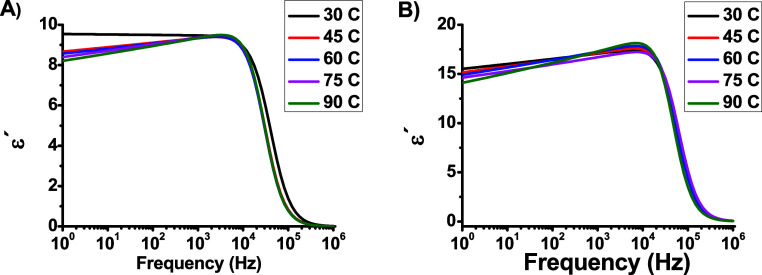
Frequency dependence of ε′ for (a) DCa1 and (b) DCa2.

**Figure 7:**
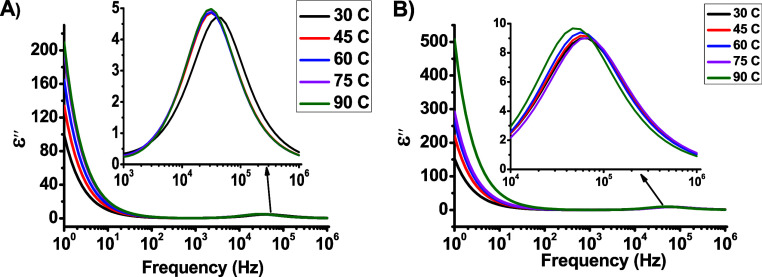
Frequency dependence of ε″ for (a) DCa1 and (b) DCa2.

The ε^′^(ω) and ε^″^(ω) can be written as^45^:
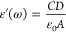
6
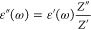
7Where C is the capacitance, D is the sample thickness, A is the cross-sectional area of the sample, and ε_0_ is the permittivity of vacuum. The frequency dependences of both ε^′^ and ε^″^ at the selected temperatures for the dot capacitors are shown in Figures 6 and 7. As shown in Figures 6a and 6b, the high permittivity of the DCa2 sample was attributed to the increased thickness of the epoxy layer, which enhanced the density of the dipoles. In cold-field emission, the thickness of the epoxy layer increases the density of the electron traps, allowing more electrons to be stored. However, this leads to longer conductive pathways, requiring a higher applied voltage to release these trapped electrons, which, in turn, increases the emission current. If the thickness of the epoxy layer is further increased, electrons may become trapped deeper within the material, requiring a higher voltage to release them. This deviates from the principle of electron emissions. The effect of the coating layer has been extensively studied and reported in literature.^13,19,46,47^

At frequencies *f* > 10^4^*Hz,* the values of ε^′^ remain constant because they are governed by the number of orientable dipoles and their ability to align under an applied electric field. For frequencies *f* < 10^4^*Hz*, the values of ε^′^ decreased with increasing frequency owing to the inability of the polarons to align with the electric field. In addition, a minor decrease in the electrical permittivity value with increasing temperature was observed, which was attributed to an increase in the number of freely moving charge carriers in the bulk region.

In Figures 7a and 7b, it can be observed that at lower frequencies, the value of ε^″^ increases with increasing temperature but decreases rapidly at higher frequencies. This behavior indicates steady-state electrical conduction of charge carriers between the electrodes. In addition, beta relaxation was dominant at higher frequencies, and a slight shift in the relaxation peak to lower frequencies was observed. This suggests that the relaxation time increased, implying that the dipoles required more time to return to their original positions after the electric field was removed. This indicates the expansion of the dipoles and formation of conducting channels, which play an important role in the emission of electrons from the sharp tip.^13,19^

### 4.3 Conductivity

The total conductivity σ_*t*_ as a function of the angular frequency (ω) can be obtained based on its dependence on the DC conductivity (σ_*DC*_) and AC conductivity (σ_*AC*_), which can be written as
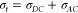
8σ_*DC*_ plays an important role in understanding the activation energy, whereas σ_*AC*_ plays an important role in the conduction mechanism.

#### 4.3.1 DC Conductivity and Activation Energy

**Figure 8:**
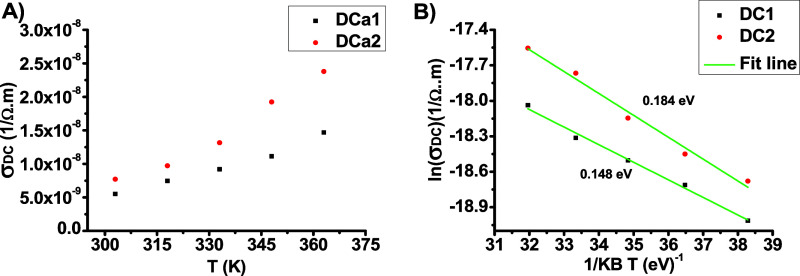
(A) Relationship between σ_DC_and T. (B) Relationship between ln(σ_DC_) and 1/*K*_*B*_*T*.

The σ_*DC*_ prevails at low frequencies and can be calculated using Ohm’s law as follows:
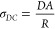
9where D is the sample thickness, A is the cross-sectional area, and R is total resistance. Furthermore, σ_*DC*_ can be expressed using the Arrhenius equation as follows^36^:
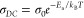
10Where σ_0_is the pre-exponential factor, *E*_*a*_ is the activation energy, *k*_*B*_ is the Boltzmann’s constant, and T is the absolute temperature. Figure 8a shows that the σ_*DC*_ increased with increasing temperature. This increase in σ_*DC*_ conductivity is due to thermal activation, which results in the release of more polarons and a higher number of mobile charge carriers in the bulk region. The temperature dependence of σ_*DC*_ was also found to follow the Arrhenius equation, as illustrated in Figure 8. The slope in Figure 8b represents the activation energy, indicating that increasing the thickness of the coating material led to a higher activation energy. This is because the densities of traps and polarons are directly proportional to the thickness of the coating layer, which requires more energy to release electrons and polarons.^13^ Specifically, the activation energy of DCa1 was 0.148 eV, whereas that of DCa2 was 0.184 eV. This finding is significant for understanding electron emission from composite cold emitters, as reported in previous studies.^1,48−51^

#### 4.3.2 AC Conductivity AC Conductivity

AC conductivity encompasses all dissipation processes, including effective ohmic conductivity, which arises from the migration of charge carriers across interfaces or within isolated semiconductor nanoparticles as well as frequency-dependent dipole scattering.^52^ AC conductivity was calculated using the following formula^53^:
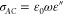
11

**Figure 9:**
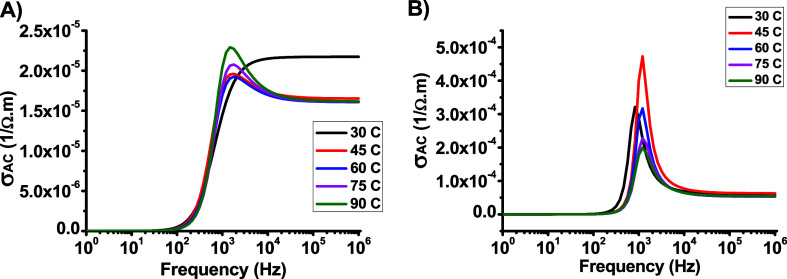
Frequency dependence of σ_AC_ for (a) DCa1 and (b) DCa2.

**Figure 10:**
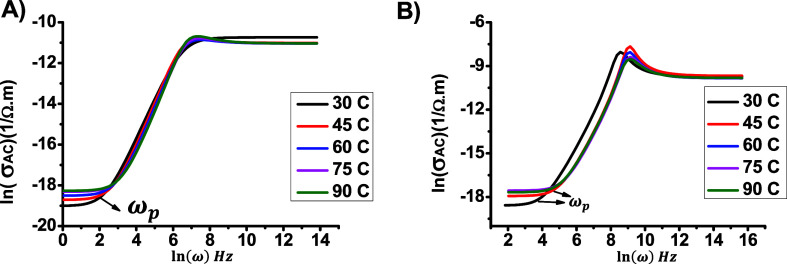
Relationship between ln(σ_AC_) and ln(ω) for (a) DCa1 and (b) DCa2.

Figures 9a and 9b show the frequency dependence of the AC conductivity (σ_*AC*_) of both the samples. The results indicate that the conductivity is temperature-independent, which can be attributed to the small size of the composite tip. Moreover, the data revealed three distinct regions that characterized the frequency dependence of σ_*AC*_. At frequencies *f* > 10^2^*Hz*, and *f* > 10^4^*Hz*, the σ_*AC*_ was frequency-independent, maintaining a constant value corresponding to the DC conductivity. The second region, known as the dispersion region (from (10^2^ – 10^3^*Hz*), begins at the crossover frequency (ω_*p*_ = 2π*f*_*p*_). where the dielectric behavior of the sample shifts from σ_*DC*_ to σ_*AC*_ y. This behavior is typical for many polymer-based samples.^22,23^ and the values of ω_*p*_ remained almost constant, as shown in Figures 10a and 10b.

The third region occurs at frequencies higher than 10^3^, where σ_*AC*_ exhibits strong frequency dependence (dispersion region). Additionally, the frequency dependence of σ_*AC*_ can be analyzed using the universal law of conductivity. Studying the temperature dependence of exponent s is crucial, as it helps determine the conduction mechanism. The values of s, which represent the slopes, are shown in Figures 10a and 10b.

**Figure 11:**
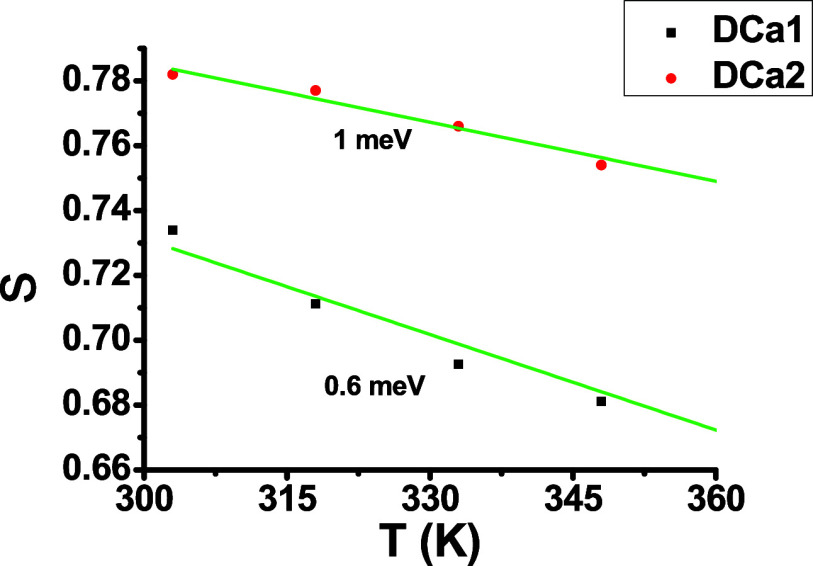
Relationship between *s* and *T*.

In addition, the conduction behavior can be considered based on the law of universality of σ_*AC*_, which is expressed as follows^54^:
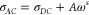
12Where A and S are dependent on the temperature and the constituent materials of the system.^55,56^Figure 11 shows the relationship between s and temperature, and it is clear that s values decrease with temperature. Based on this information, the conduction mechanism can be explained using the modified Coherent Barrier Hopping (CBH) model. The CBH model explains the hopping mechanism of charge carriers (or polarons) in pairs between localized sites at the Fermi level via current transport in a system, and according to the negative dependence of S on temperature.^57,58^ In the modified CBH model, S is expressed as^59^
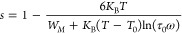
13where τ_0_ = 10^–13^*s* represents the relaxation time of the atomic vibrations and *T*_0_ is the temperature at which S is unity. The CBH model in terms of AC conductivity is expressed as follows^60^:
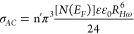
14where n′ is the number of polarons or charge carriers involved in the hopping process, *N* (*E*_*F*_)is the concentration of pair states, *R*_*H*ω_is the hopping distance at frequency ω,, and. The bipolar hopping process was assumed to be the dominant conduction mechanism. Based on fitting data on Figure 11, the *W*_*M*_ for DCa1 was 0.6 meV, while for DCa2 it was 1 meV, respectively, owing to the thicker epoxy layer. This increase in *W*_*M*_ is likely due to the greater interference between the potential wells within the epoxy layer, as described by Pike’s model.^59^ Based on the low WM values, it can be said that the movement of charge carriers is driven by short-term jumps across the Coulomb barrier that separate the two defect centers. This is different from DC conduction, in which the movement of carriers is characterized by long-range jumps. This makes epoxy an effective material for field emission applications and transistors that require the application of several hundred volts of free electrons from the surface of the composite emitter.^36^

### 4.4 Nyquist and Cole–Cole plots

This equation relates the real and imaginary parts of the impedance to examine the changes in series resistance (*R*_*s*_), paralel resistance (*R*_*p*_) and capacitance under varying frequency and temperature conditions. The semicircles observed in the impedance plot are centered below the real axis, whereas the ideal semicircle is centered on the real axis and intersects at low frequencies. The ideal half-circuit behavior can be effectively modeled by a parallel RC circuit, where the bulk resistance of the sample is estimated from the radius of the semicircle.^61^

**Figure 12:**
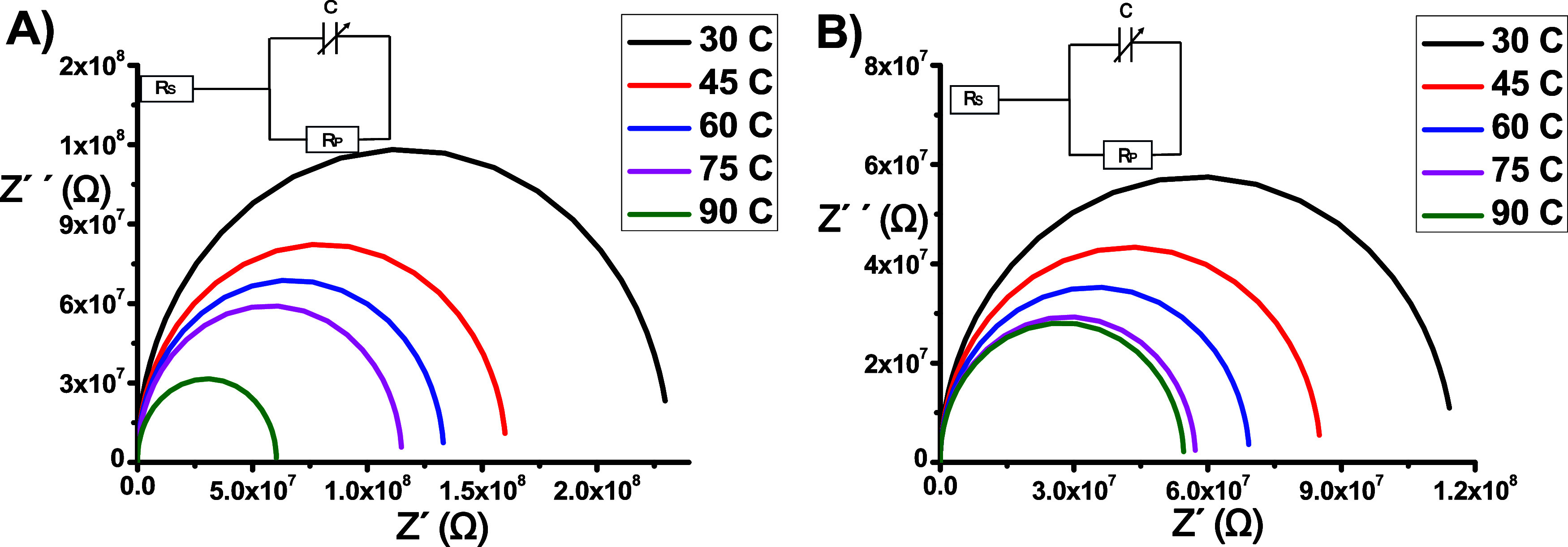
Nyquist plots for (A) DCa1 and (B) DCa2.

Deviations from ideal capacitive behavior can be modeled by introducing a constant phase element (CPE) into the circuit to replace the capacitor. The Nyquist equation and impedance of the CPE element are given by^45,62^
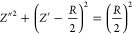
15
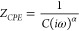
16Where C is the capacitance value of the CPE element and α represents the deviation from Debye’s model (0 < α < 1). As α approaches 1, CPE behaves like an ideal capacitor.^63^ Nyquist plots for both samples at the selected temperatures are shown in Figures 12a and 12b. Parameters *R*_*s*_, *R*_*p*_, C, and α are listed in Table 1. values are close to the ideal state. However, the values of Rs, Rp, and C decreased with increasing temperature, indicating that the charge-carrier diffusion was thermally activated. The inverse relationship between the total resistance and temperature suggests an increase in electrical conductivity and a decrease in relaxation time, which is characteristic of typical semiconductor behavior.

**Table 1:** Values of the Parameters R1, R2, *Q*, and α

	*R*_s_ (Ω)		*R*_p_ (Ω)		*C* (F)		**α**	
*T* (*°*C)	DCa1	DCa2	DCa1	DCa2	DCa1	DCa2	DCa1	DCa2
30	3.5 × 10^5^	7 × 10^5^	1.2 × 10^8^	1.2 × 10^8^	7.4 × 10^–11^	6.6 × 10^–11^	0.97	0.98
45	9.4 × 10^4^	1.2 × 10^5^	8.5 × 10^7^	8.5 × 10^7^	8.2 × 10^–12^	2.4 × 10^–11^	0.98	0.97
60	8.4 × 10^4^	9.2 × 10^4^	7 × 10^7^	7 × 10^7^	6.8 × 10^–12^	9.7 × 10^–12^	0.96	0.95
75	7.3 × 10^4^	8.3 × 10^4^	5.7 × 10^7^	5.7 × 10^7^	5.2 × 10^–12^	7.9 × 10^–12^	0.93	0.96
90	5.5 × 10^4^	6.7 × 10^4^	5.4 × 10^7^	5.4 × 10^7^	3.3 × 10^–12^	6.4 × 10^–12^	0.95	0.98

When comparing the impedance and capacitance values of both samples, we noticed that the values for DC2 were higher than those for DC1. This result is important for explaining the emission of electrons from composite emitters reported in the literature.^12,13,64^ The thicker the coating material, the higher the total impedance; thus, more voltage must be applied to release electrons. In addition, increasing the thickness of the coating material leads to an increase in the capacitance, and when the applied voltage reaches the turn-on voltage, the capacitance is broken down. Thus, the current value of the composite emitters is higher than that of the uncoated emitters.

**Figure 13:**
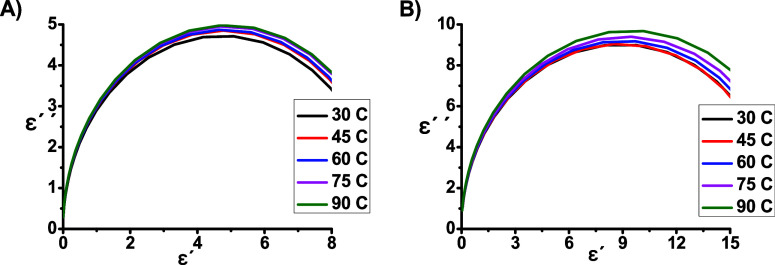
Cole–Cole plots for (A) DC1 and (B) DC2.

Figure 13b presents the Cole–Cole plot, which shows a slight increase in the radius of the semicircle, indicating a minimal effect of temperature on dipole rotation at low frequencies. At higher frequencies, the material exhibited conductive behavior owing to the accumulation of charge at the electrodes.^19^

The relaxation properties have primarily been studied using the Debye relaxation model, which describes the dielectric relaxation response of an idealized, noninteracting set of dipoles in an external alternating electric field. The Debye relaxation model was modified by Havriliak- Negami by introducing two exponential parameters into the original Debye equation. Unlike the Debye model, the H–N relaxation model accounts for the asymmetry and amplitude of the electric-dispersion curve. This modified model has been effectively used to describe the dielectric relaxation of various polymers, and can be expressed as follows^65^:
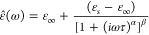
17where ε_∞_ is the optical permittivity, ε_*s*_ is the static permittivity, α is the maximum flatness/width, and β is the skewness of the complex dielectric permittivity. The Cole–Cole equation is a special case of the HN function when the symmetry parameter β is equal to 1 and 0 < α < 1, that is, when the relaxation peaks are symmetric and can be expressed by the following equation^66^:
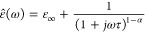
18When α equals zero, the Cole–Cole model simplifies to the Debye model. By contrast, α values greater than zero indicate that the relaxation process is dispersed across a broad spectrum of frequencies. The Cole–Cole plot is a valuable tool for investigating the frequency-dependent behavior of the complex permittivity in dielectric materials. This plot represents a graph of the real (ε′) and imaginary (ε″) components of the complex permittivity versus the frequency parameter. Figure 13 shows the Cole–Cole plots of the point capacitance at selected temperatures.

As shown in Figure 13, the center of the asymmetric semicircle is located below the *x*-axis, indicating that the relaxation process does not conform to the Debye model. Figures 13a and 13b reveal a slight increase in the radius, suggesting a minor increase in the relaxation time caused by the heating process, which is attributed to the small size of the fluid. Furthermore, this observation indicates that the breakdown of the epoxy layer, resulting from the formation of conductive channels in the cold-field emitters, is due to the heating effect produced by the application of voltage to the emitter. These results are consistent with previously published findings.^19^

## Conclusions

The results of this study demonstrate that the composite microtip behaves as a dot capacitor. It exhibited stable performance across varying temperatures, likely owing to its compact size. The results show that variations in the tip radius and epoxy layer thickness significantly affect the impedance, permittivity, and conductivity, enhancing both the coating layer and the electrode and the dipole density. A minor decrease in both the impedance and permittivity was observed with increasing temperature, indicating the semiconducting nature of the epoxy. Moreover, the AC conductivity of the dot capacitor did not exhibit temperature dependence. These results indicate that the activation energy of the dot capacitors is relatively low. Furthermore, the conduction mechanism was described by a modified coherent barrier-hopping model, and it was found that the barrier voltage was low. This makes it easy for an electron to jump between two defect centers or tunnel through conductive pathways in the epoxy layer when exposed to a few hundred volts. The Nyquist plot shows a small decrease in point capacitance with increasing temperature. In addition, the Cole–Cole plot indicated a minor increase in the relaxation time, suggesting the emergence of conductive pathways. Therefore, the results of this study pave the way for further research in this area, such as testing composite nanostructured emitters and investigating how voltage affects the insulating properties of the coating material.
